# Cerebral large artery stenosis and occlusion in POEMS syndrome

**DOI:** 10.1186/s12883-021-02260-2

**Published:** 2021-06-24

**Authors:** Atsuhiko Sugiyama, Hajime Yokota, Sonoko Misawa, Hiroki Mukai, Yukari Sekiguchi, Kyosuke Koide, Tomoki Suichi, Jun Matsushima, Takashi Kishimoto, Zen-ichi Tanei, Yuko Saito, Shoichi Ito, Satoshi Kuwabara

**Affiliations:** 1 Department of Neurology, Graduate School of Medicine, Chiba University, 1-8-1 Inohana, Chuo-ku, 260-8677 Chiba, Japan; 2 Department of Diagnostic Radiology and Radiation Oncology, Graduate School of Medicine, Chiba University, Chiba, Japan; 3 Department of Radiology, Chiba University Hospital, Chiba, Japan; 4 Department of Neurology, JR Tokyo General Hospital, Tokyo, Japan; 5 Department of Pathology, Saitama Medical Center, Dokkyo Medical University, Saitama, Japan; 6 Department of Diagnostic pathology, Graduate School of Medicine, Chiba University, Chiba, Japan; 7 Department of Molecular Pathology, Graduate School of Medicine, Chiba University, Chiba, Japan; 8 Department of Pathology and Laboratory Medicine, National Center of Neurology and Psychiatry, Tokyo, Japan; 9 Department of Medical Education, Graduate School of Medicine, Chiba University, Chiba, Japan

**Keywords:** Magnetic resonance angiography, Computed tomography angiography, Cerebral infarction, Vasospasm, Castleman disease, Vascular endothelial growth factor

## Abstract

**Background:**

This study aimed to investigate the frequency and risk factors for cerebral artery stenosis and occlusion in patients with polyneuropathy, organomegaly, endocrinopathy, M-protein, and skin changes (POEMS) syndrome.

**Methods:**

We reviewed results of magnetic resonance angiography (MRA) or computed tomography angiography (CTA) in 61 patients with POEMS syndrome seen between 2010 and 2017. Stenosis or occlusion was assessed in the initial MRA/CTA. Multivariate analysis was used to identify risk factors for artery stenosis/occlusion. In an autopsy case, pathologic examination was conducted of the occluded middle cerebral arteries.

**Results:**

Stenosis (> 50 %) or occlusion of the major cerebral arteries was found in 29 (47.5 %) patients on the initial MRA/CTA. The internal carotid artery was involved most frequently (32.8 %), followed by the anterior (21.3 %) and middle (16.4 %) cerebral arteries. The basilar (1.3 %) and vertebral (3.6 %) arteries were rarely affected. Cerebral infarction developed in eight (13.1 %) patients. The serum vascular endothelial growth factor (VEGF) level was an independent predictor for stenosis/occlusion (odds ratio, 1.228; 95 % confidence interval, 1.042–1.447; *P* = 0.014). An autopsy study showed occluded middle cerebral arteries by fibrous and myxomatous thickening of intima with splitting of the internal elastic lamina. Follow-up MRA in 23 patients showed improved, worsened, and unchanged stenosis in 20.7 %, 8.7 %, and 69.6 %, respectively.

**Conclusions:**

Cerebral large-vessel stenosis or occlusion is frequently seen in approximately half of patients with POEMS syndrome. Vasculopathy was related to serum VEGF levels and thereby disease activity. Assessment of cerebral vessels is recommended in these patients to improve management.

**Supplementary Information:**

The online version contains supplementary material available at 10.1186/s12883-021-02260-2.

## Background

Polyneuropathy, organomegaly, endocrinopathy, M-protein, and skin changes (POEMS) syndrome is a rare multisystemic disease associated with plasma cell dyscrasia [1]. Upregulated vascular endothelial growth factor (VEGF) or other inflammatory cytokines presumably have a major pathogenic role for the development of clinical manifestations, such as capillary leak syndrome (pleural effusion, ascites, and edema), skin angioma, and possibly peripheral neuropathy [2]. Particularly, serum VEGF levels correlated with disease activity [1, 3].

Whereas peripheral neuropathy is a core manifestation of the disorder, central nervous system involvement in POEMS syndrome has not been sufficiently investigated. However, previous studies have described stroke, papilledema, and pachymeningeal involvement in patients with POEMS syndrome [4–7]. One case series suggested that brain infarction is not a rare complication with a 13.4 % overall 5-year risk [5]. Although the mechanisms underlying brain infarction in POEMS syndrome remain unknown, thrombocytosis, polyglobulia, hyperfibrinogenemia, and high circulating pro-inflammatory cytokine levels are potential factors, which trigger a hypercoagulatory state [1, 5, 8].

Major cerebral vessel stenosis or occlusion and ischemic stroke have been reported in patients with POEMS syndrome [5, 6]. Cerebral infarction has also been reported in a Castleman disease variant of POEMS syndrome [9], and a correlation with cerebral vasculopathy and Castleman disease in patients with POEMS syndrome has been described [10, 11]. However, to the best of our knowledge, no large study exists on cerebral vessels in POEMS syndrome, and the frequency and nature of cerebral vasculopathy remain unclear.

We therefore studied systematically the frequency and features of changes in large cerebral arteries in patients with POEMS syndrome.

## Methods

Any data not published within the article will be anonymously shared upon request from any qualified investigator.

### Subjects

Of 75 consecutive patients with POEMS syndrome screened at Chiba University Hospital between January 2010 and July 2017, 61 were included in this study. Inclusion criteria were fulfilling the recently proposed diagnostic criteria for POEMS syndrome [12] and sufficient data available on cerebral computed tomography angiography (CTA) or magnetic resonance angiography (MRA). One patient had the Castleman disease variant of POEMS syndrome [1].

Medical records were reviewed for clinical and laboratory data. Relevant comorbidities, such as cerebral infarction, Castleman disease, and pulmonary hypertension, were reviewed from onset of POEMS syndrome to the last follow-up by January 2018. Other clinical and laboratory data ware reviewed up to the initial MRA/CTA. Hypertension was defined as taking antihypertensive drugs, diabetes as taking antidiabetic agents or a hemoglobin A1c ≥6.5 %, and dyslipidemia as taking antihyperlipidemic drugs and total, low-density, or high-density cholesterol levels of ≥ 220, ≥140, or < 40 mg/dL, respectively. Smoking was defined as regular smoking within the last 3 years. Serum VEGF levels were measured by enzyme-linked immunosorbent assay, as previously described [12].

This study procedure was approved by the institutional review board of the Chiba University Graduate School of Medicine, and the need for informed consent was waived. Additional written informed consent was obtained for our reported case.

### Image analysis on cerebral arteries

Two experienced neuroradiologists (H.Y. and H.M.), who were blinded to the clinical data, evaluated the initial MRA/CTA of each subject. Intracranial vasculature evaluated included the anterior cerebral (ACA), middle cerebral (MCA), vertebral (VA), posterior cerebral (PCA), internal carotid (ICA), and basilar (BA) arteries. The degree of cerebral artery stenosis was visually assessed as grade 0 (0–50 % of the vascular diameter), grade 1 (51–99 % of the vascular diameter or a significant stenosis with residual flow signal downstream), and grade 2 (occlusion or a vascular amputation without residual flow signal downstream). Arterial stenosis was graded by comparing the diameters of the maximally stenosed artery and the more proximal normal segment of the same vessel [13]. Grade 1 or 2 was defined as a significant lesion. In 23 patients in whom MRAs were performed several times, the latest MRA performed by January 2018 was also evaluated in each patient as a follow-up and compared to the initial MRA. The change in the MRA findings were defined as follows: improved, showed a decrease of ≥ 1 point in the grade of stenosis at any region of the intracranial arteries; worsened, showed an increase of ≥ 1 point in the grade of stenosis at any region of the intracranial arteries.

### Statistical analysis

All statistical analyses were performed using SPSS software, ver. 25.0 (SPSS, Tokyo, Japan). Demographic and clinical variables between patients with and without stenosis/occlusion were compared using the Student’s *t*-test and Mann–Whitney *U* test for continuous variables and the χ^2^ and Fisher’s exact probability tests for categorical variables. Variables with *P* < 0.1 after univariate analysis were included in a multivariate analysis by logistic regression to identify the independent predictor for cerebral artery stenosis/occlusion on MRA/CTA in POEMS syndrome. A forward stepwise logistic regression analysis, with the likelihood method (probability for stepwise, *P* < 0.05 for entry and *P* < 0.10 for removal), was used. *P* < 0.05 was considered statistically significant. Weighted κ statistics were calculated to assess inter-rater agreements.

## Results

Table 1 shows the demographics of patients with POEMS syndrome and the Castleman disease variant. Median time from onset to final follow-up by January 2018 was 5.8 (range, 1.6–18.1) years. Cerebral infarction was observed in eight patients (13.1 %; one asymptomatic and seven symptomatic). Of the eight patients, cerebral infarction was confirmed in five patients at the time of initial MRA and the remaining three patients after the initial MRA/CTA. Only one of the eight patients with cerebral infarction did not show stenosis/occlusion on MRA/CTA. In the remaining seven patients, cerebral infarction was observed in the territory of cerebral artery in which stenosis/occlusion was demonstrated on MRA/CTA.

**Table 1:** Comparison of patients with and without cerebral artery stenosis/occlusion in POEMS syndrome

Characteristics	All (*n* = 61)	Stenosis/occlusion (*n* = 29)	No stenosis/occlusion (*n* = 32)	*P* value
Demographics				
Age at MRA/CTA, y, (mean ± SD) ^a^	55.5 ± 13.1	53.2 ± 14.9	57.4 ± 11.1	0.210
Sex (male/female) ^b^	36/25	16/13	20/12	0.561
Onset to diagnosis, y, median (range) ^c^	1.5 (0.2–10.3)	1.4 (0.2–10.3)	1.6 (0.2–9.6)	0.806
Onset to MRA/CTA, y, median (range) ^c^	2.7 (0.2–13.3)	2.4 (0.2–12.4)	2.9 (0.2–13.3)	0.948
Relevant comorbidities, n (%)				
Cerebral infarction ^d^	8 (13.1)	8 (27.6)	0 (0)	**0.001**
Castleman disease ^d^	8 (13.1)	3 (10.3)	5 (15.6)	0.412
Pulmonary hypertension ^d^	4 (6.6)	3 (10.3)	1 (3.1)	0.270
Relevant POEMS features, n (%)				
Polyneuropathy	61 (100)	29 (100)	32 (100)	NA
Organomegaly ^d^	55 (90.2)	26 (90.0)	29 (90.6)	0.616
Endocrinopathy ^b^	37 (60.7)	19 (65.5)	18 (56.3)	0.459
Extravascular volume overload ^d^	59 (96.7)	29 (100)	30 (93.8)	0.271
Monoclonal plasma cell proliferative disorder ^d^	60 (98.4)	28 (96.6)	32 (100)	0.475
Skin changes ^d^	59 (96.7)	28 (96.6)	31 (96.9)	0.729
Sclerotic bone lesions ^d^	52 (85.2)	23 (79.3)	29 (90.6)	0.189
Laboratory studies				
Maximum VEGF value, ng/ml, median (range) ^c^	4.910 (1.03–31.70)	7.250 (1.33–31.70)	4.695 (1.03–11.70)	**0.028**
Risk factors for atherosclerosis, n (%)				
Hypertension ^b^	23 (37.7)	10 (34.5)	13 (40.6)	0.621
Dyslipidemia ^b^	27 (44.3)	14 (48.3)	13 (40.6)	0.548
Diabetes ^d^	7 (11.5)	4 (13.8)	3 (9.4)	0.443
Smoking ^d^	8 (13.1)	4 (13.8)	4 (12.5)	0.588
Previous treatment, n (%)				
None ^b^	31 (50.8)	12 (41.4)	19 (59.4)	0.160
Corticosteroids ^b^	31 (50.8)	17 (58.6)	14 (43.8)	0.246
Melphalan ^d^	6 (9.8)	3 (10.3)	3 (9.4)	0.616
Cyclophosphamide ^d^	2 (3.3)	2 (6.9)	0 (0)	0.222
Thalidomide ^b^	13 (21.3)	9 (31.0)	4 (12.5)	0.078
ASCT ^d^	9 (14.8)	6 (20.7)	3 (9.4)	0.189
Renalidomide ^d^	1 (1.6)	1 (3.4)	0 (0)	0.475
Bortezomib ^d^	1 (1.6)	1 (3.4)	0 (0)	0.475
Bevacizumab ^d^	2 (3.3)	2 (6.9)	0 (0)	0.222
Radiation ^d^	3 (4.9)	1 (3.4)	2 (6.3)	0.537

*MRA* magnetic resonance angiography; *CTA* computed tomography angiography; *NA* not applicable; *ASCT* autologous peripheral blood stem-cell transplantation

^a^Student’s *t*-test

^b^χ2 test

^c^Mann–Whitney *U* test

^d^Fisher’s exact probability test

### Evaluation of the initial MRA/CTA

All patients underwent brain MRA, except one who underwent only CTA. MRA was performed on a 1.5-T MRI system in 44 patients and a 3-T system in 16. There was no statistically significant difference in type of MRI system between the stenosis/occlusion and no stenosis/occlusion groups (*P* = 0.785). The κ value for inter-rater agreement between the two examiners was 0.739.

**Fig. 1:**
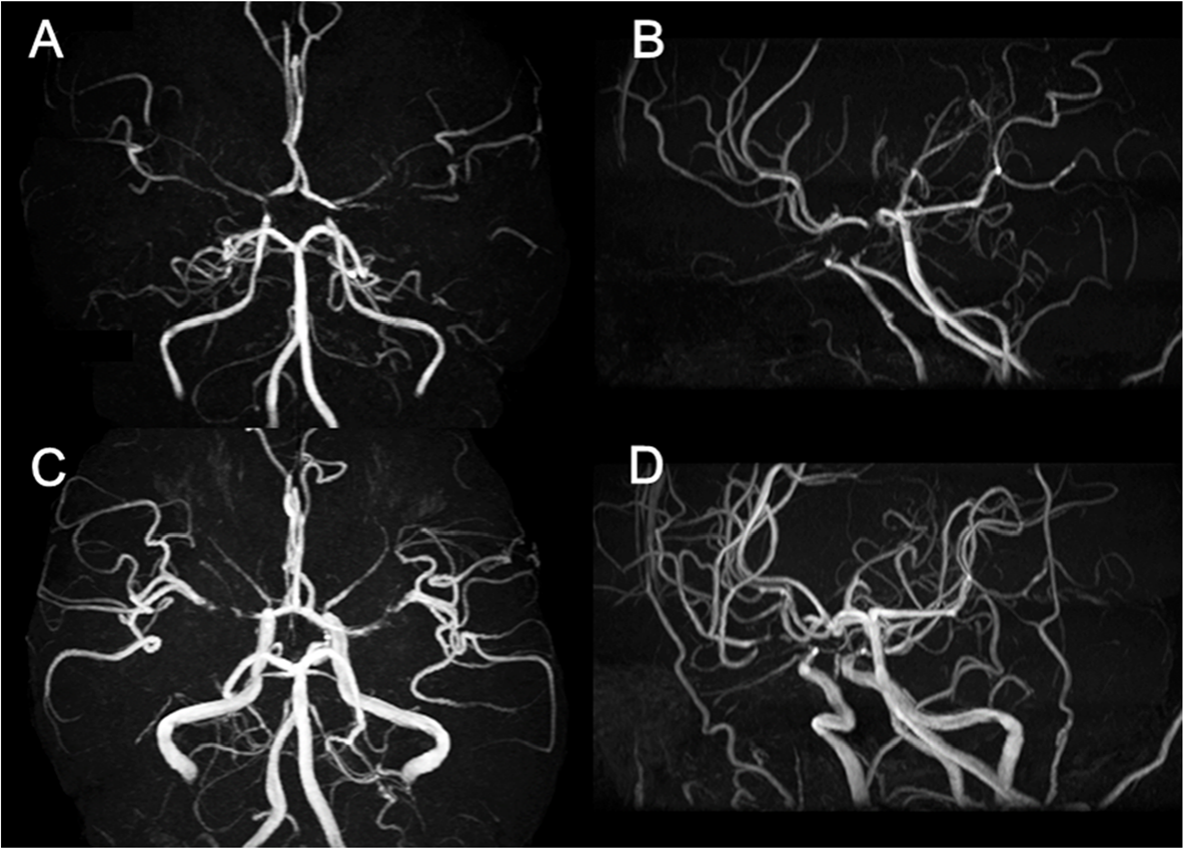
Brain magnetic resonance angiography (MRA) of a patient with Castleman disease variant of POEMS syndrome. Brain MRA (**A**, **B**) of a 16-year-old man revealed stenosis (Grade 1) of the proximal portion of the bilateral anterior cerebral arteries (ACAs) and middle cerebral arteries (MCAs), and the terminal portion of the internal carotid arteries (ICAs). Diagnosis was Castleman disease variant of POEMS syndrome, and bortezomib and dexamethasone therapy was started. Although the bilateral ACAs, MCAs, and ICAs stenosis remained, the flow signal of the cerebral arteries was thickened overall on follow-up MRA 22 months after the initial MRA (**C**, **D**)

**Fig. 2:**
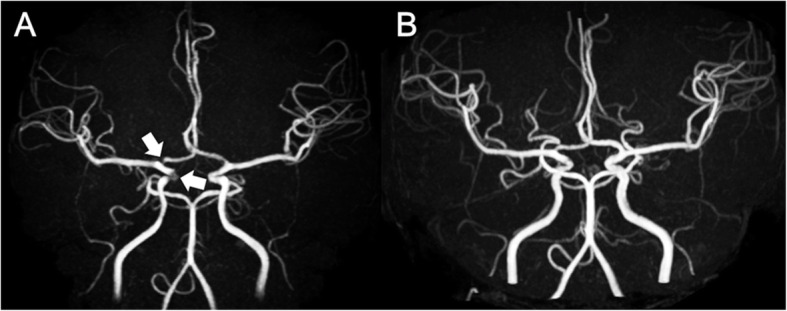
A patient with POEMS syndrome in whom stenosis improved on follow-up magnetic resonance angiography (MRA). Brain MRA (**A**) of a 34-year-old woman revealed stenosis (Grade 1) of the right anterior cerebral artery (ACA) and internal carotid artery (ICA). Diagnosis was POEMS syndrome and treatment began with thalidomide and dexamethasone, followed by lenalidomide and dexamethasone, and autologous peripheral blood stem-cell transplantation. Although the right ACA stenosis remained, the right ICA stenosis was improved and the flow signal of the cerebral arteries was thickened overall on follow-up MRA (**B**) 30 months after the initial MRA

**Fig. 3:**
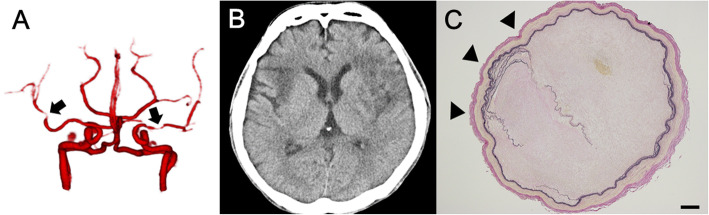
Computed tomography angiography (CTA), computed tomography (CT), and pathological finding of the autopsy case. CTA at 6.8 years after diagnosis of POEMS syndrome (**A**), when the patient presented with recurrent left hemiplegia, showed stenosis (Grade 1) in the bilateral middle cerebral arteries (MCAs) (*black arrows*) and left carotid arteries and anterior cerebral arteries. CT 9 weeks later (**B**) revealed extensive infarction in the bilateral MCA territory. Autopsy demonstrated MCA was occluded and Elastica van Gieson (EVG) staining showed disruption and duplication of the internal elastic lamina (*black arrowheads*) (**C**). Scale bars: (**C**) 200 μm

Cerebral MRA/CTA showed significant lesions (Grade 1 or 2) in 29 (47.5 %) of 61 patients (Table 2). Among the major vessels, the ICA, ACA, and MCA were frequently involved, whereas posterior circulation (VA, BA, and PCA) involvement was rare. Multiple vessel involvement was observed in 21 (34.4 %) patients (Figs. 1, 2 and 3). Three of 29 (10.3 %) patients with stenosis/occlusion developed cerebral infarction from the time of the initial MRA/CTA to the final follow-up in January 2018.

**Table 2:** Number and site of cerebral artery stenoses/occlusions in POEMS syndrome

	Stenosis ^a^	Occlusion ^b^	Stenosis or occlusion
	(*n* = 26)	(*n* = 5)	(*n* = 29)
% in 61 patients	26/61 (42.6 %)	5/61 (8.2 %)	29/61 (47.5 %)
Site of MRA/CTA lesions			
Internal carotid artery	17/61 (27.9 %)	3/61 (4.9 %)	20/61 (32.8 %)
Anterior cerebral artery	12/61 (19.7 %)	1/61 (1.6 %)	13/61 (21.3 %)
MCA	8/61 (13.1 %)	2/61 (3.3 %)	10/61 (16.4 %)
BA	1/61 (1.6 %)	0/61 (0 %)	1/61 (1.6 %)
VA	1/61 (1.6 %)	1/61 (1.6 %)	2/61 (3.3 %)
PCA	4/61 (6.6 %)	1/61 (1.6 %)	5/61 (8.2 %)

Data are expressed as number (%); *MRA* magnetic resonance angiography; *CTA* computed tomography angiography

^a^51–99 % of the vascular diameter or a significant stenosis with residual flow signal downstream

^b^Occlusion or a vascular amputation without residual flow signal downstream

### Univariate and multivariate analyses

The patient groups with (*n* = 29) and without (*n* = 32) stenosis/occlusion on the initial MRA/CTA are compared in Table 1. Maximum serum VEGF level was significantly higher in patients with significant lesions on MRA/CTA than in those without. No other clinical and laboratory profiles were significantly different in the two patient groups.

For variables with *P* < 0.1 after univariate analysis, the maximum serum VEGF level and previous thalidomide treatment were included in a multiple regression analysis. Cerebral infarction was considered to be a result rather than a cause of cerebral artery stenosis/occlusion; therefore, it was not included in a multiple regression analysis as a variable. A stepwise forward method generated a model that included maximum serum VEGF level as the variable and found this level to be the independent predictor for cerebral artery stenosis/occlusion on MRA/CTA (odds ratio [OR], 1.228; 95 % confidential interval [CI], 1.042–1.447; *P* = 0.014). The model was significant (χ^2^ = 8.334, 1 degree of freedom, *P* = 0.004), and the Hosmer–Lemeshow χ^2^ goodness-of-fit statistic was nonsignificant (*P* = 0.111). Nagelkerke and Cox-Snell *R*^2^ values were 0.128 and 0.170, respectively. The model accurately identified 72.1 % of patients with POEMS and cerebral artery stenosis/occlusion on MRA/CTA.

### Evaluation of the follow-up MRA

The follow-up MRA was performed on a 1.5-T MRI system in 20 patients and a 3-T system in 3. Median time from initial to follow-up MRA was 1.0 (0.1–6.0) years. The κ value for inter-rater agreement between the two examiners was 0.779. Compared to the initial MRA, the findings on follow-up MRA were improved, worsened, and unchanged in 5 (21.7 %), 2 (8.7 %), and 16 (69.6 %) patients, respectively (Fig. 2). There were no patients for whom both improved and worsened regions were observed on the follow-up MRA. Detailed clinical data of the five patients with improved MRA findings are summarized in Additional file 1. Serum VEGF level was markedly decreased at the time of follow-up MRA compared to that at the initial MRA in all five patients with improved MRA findings.

### Autopsy case report

A 43-year-old Japanese man with a history of hypertension presented to our hospital with a 15-month history of hypertrichosis and a 9-month history of numbness in the toes. He was diagnosed with POEMS syndrome based on polyneuropathy, organomegaly, M protein, skin changes, and elevated serum VEGF level (7160 pg/mL). Four months after diagnosis, he underwent autologous peripheral blood stem-cell transplantation. Five years later, edema of the lower limbs and repeat elevated serum VEGF levels were observed. He was included in a randomized clinical trial, the POEMS Syndrome Thalidomide (J-POST) Trial, and was assigned to the placebo group [14]. Four months after starting the trial, he presented with ileus and improved with conservative treatment. Seven weeks later, cardiac arrest lasting for 9 s occurred and spontaneously resolved. A pacemaker was implanted.

Two weeks after implantation, he presented with recurrent left hemiplegia, which was elicited by rising from a lying posture. CTA revealed bilateral MCA stenosis (Fig. 3). Seven weeks later, he presented with consciousness disturbance and right hemiplegia. Brain CT revealed an infarction in the left corona radiata and right frontal deep white matter. Blurring of the gray-white matter junction, considered an early CT sign, was also observed in the right insula. Follow-up CT 19 days after the prior CT revealed extensive infarction in the bilateral MCA territory (Fig. 3). He died of renal failure.

**Fig. 4:**
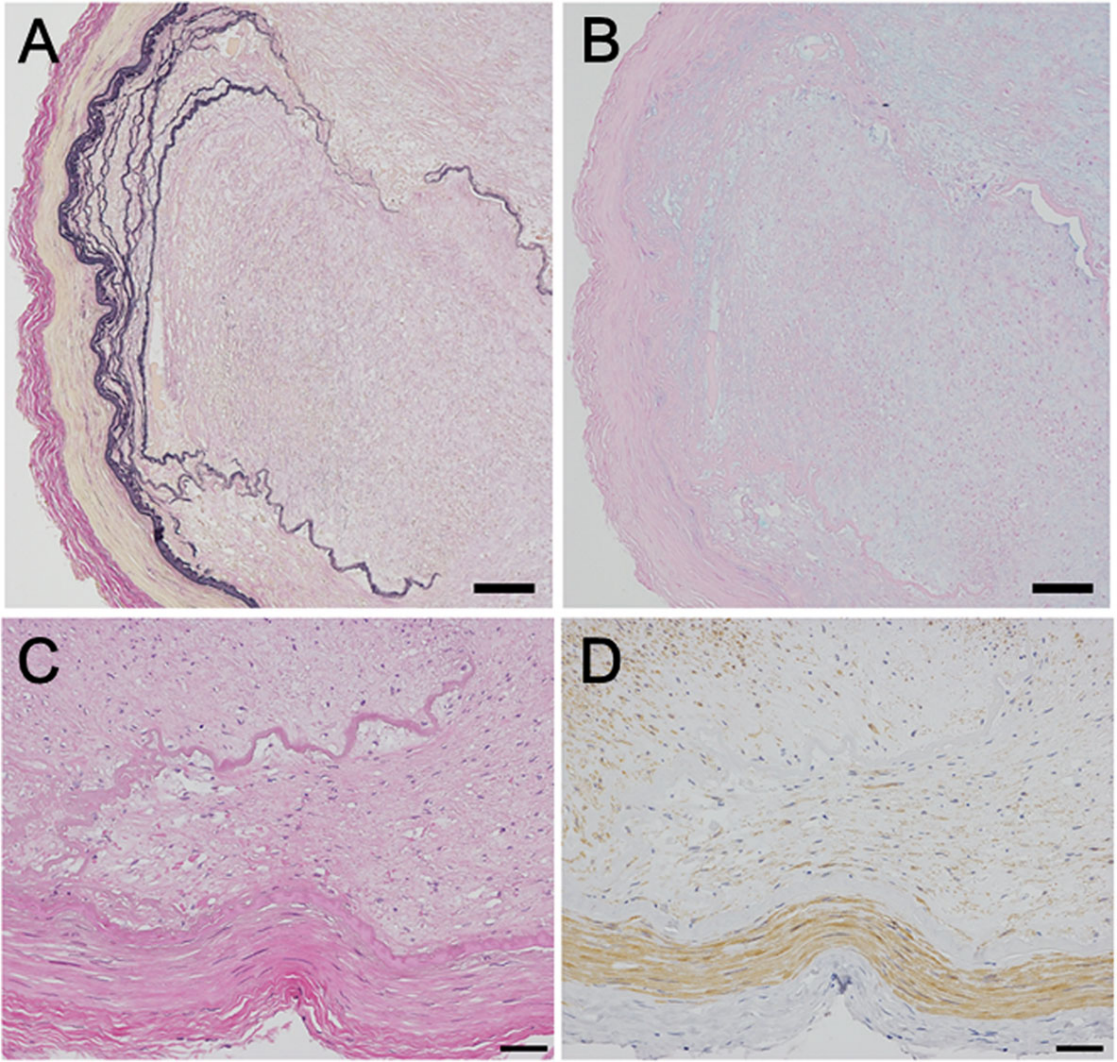
Microscopic pathological findings in the middle cerebral artery (MCA) of the autopsy case. Elastica van Gieson (EVG) staining showed disruption and duplication of the internal elastic lamina (**A**). Alcian blue staining demonstrated faint but diffuse staining of the thickened intima (**B**). High-power field of the area where disruption of the internal elastic lamina was observed (**C**, **D**). No lipid deposition or calcification suggesting atherosclerosis and no inflammatory cell infiltration were observed (hematoxylin and eosin staining, **C**). Immunochemistry with antihuman α-SMA monoclonal antibodies revealed SMA-positive spindle cells in the fibrotic intima (D). Scale bars: (**A**, **B**) 100 μm; (**C**, **D**) 50 μm

Autopsy demonstrated infarction in the bilateral MCA territories and both MCAs were occluded or severely stenosed (≥ 95 %) (Fig. 3). Microscopic studies revealed marked fibrous and myxomatous thickening of the intima with focal disruption and duplication of the internal elastic lamina. Loose fibrous tissues with spindle cells immunohistochemically positive for α-smooth muscle actin (SMA), suggesting myofibroblasts, were increased in the thickened intima and mildly stained with alcian blue. There was no lipid deposition or calcification suggesting atherosclerosis and no evidence of vasculitis, such as inflammatory cell infiltration, multinucleated giant cells, or granulomas (Fig. 4).

## Discussion

We demonstrated that about half of the patients with POEMS syndrome had stenosis/occlusion of the major cerebral arteries on the initial MRA/CTA. Multiple vessel involvement was observed in approximately one-third of all patients. In addition, multivariate logistic regression analyses showed that maximum serum VEGF was the independent predictor for cerebral artery stenosis/occlusion. An autopsy case showed severe stenosis of both MCAs with fibrous and myxomatous intimal thickening. Stenotic lesion improvement was observed in approximately one-fifth of patients who underwent a follow-up MRA.

Cerebral artery stenosis/occlusion on MRA/CTA is not a rare finding in POEMS syndrome. This condition was noted in approximately half of our patients with POEMS syndrome and the Castleman disease variant. Cerebral infarction was observed in 13.1 % of patients and was comparable in frequency to the previously reported overall 5-year risk of 13.4 % [5]. Cerebral artery stenosis/occlusion in POEMS syndrome has been previously reported [5, 6, 11, 15].

Our study also suggested that the main cause of cerebral artery stenosis/occlusion in POEMS syndrome is not atherosclerosis or inflammatory changes, but vasculopathy related to disease activity of the POEMS syndrome itself. In our study, cerebral artery stenosis/occlusion was related to maximum serum VEGF level, which presumably reflected disease activity of the POEMS syndrome. The frequency of risk factors for atherosclerosis was not different between POEMS syndrome and the Castleman disease variant among patients with and without cerebral artery stenosis/occlusion.

In our autopsy case, in which antemortem CTA revealed MCA stenosis, autopsy confirmed fibrous and myxomatous intimal thickening without apparent atherosclerotic or inflammatory changes. As with our case, a previous autopsy case with quasi-moyamoya disease associated with POEMS syndrome showed duplication of the internal elastic lamina and marked fibrous thickening localized to the intima of the MCA without evidence of vasculitis or atherosclerosis [16]. In 2017, Fu et al. reviewed 28 cases of POEMS syndrome complicated by ischemic stroke and found that 19 of 25, in which the cerebral arteries were evaluated, had stenosis/occlusion of the ICA. Eleven of these 19 cases had no risk factors for stroke [11].

Previous reports also have considered the nature of cerebral vasculopathy in POEMS syndrome as noninflammatory due to the benign manifestation of the cerebrospinal fluid [5, 17]. On the other hand, some reports have suggested that cerebral artery stenosis was due to large-vessel vasculitis [11, 15, 18], including patients who showed thickening and enhancement of the vessel wall of the cerebral artery on contrast-enhanced MRI, suggestive of vasculitis [11, 15]. In one case of POEMS syndrome with pachymeningeal involvement, since ICA stenosis was found where the ICA penetrated the dura, secondary inflammation from pachymeningitis was considered a cause of ICA stenosis [18]. However, contrast enhancement of the arterial wall was related not only to vasculitis but also to other etiologies, such as neovascularization or hyperproliferation of the vessel wall components [19, 20]. Histopathologic study of pachymeningeal involvement has shown that meningeal involvement in POEMS syndrome was due to noninflammatory changes [7].

VEGF might have a pivotal role in cerebral vasculopathy development in POEMS syndrome. In our study, multiple logistic regression showed that maximum serum VEGF level was the independent predictor for cerebral artery stenosis/occlusion on MRA/CTA in patients with POEMS syndrome. A previous autopsy report also suggested that high VEGF levels accelerate intimal thickening via ectopically existing SMA-positive smooth muscle cells [16]. In the previous autopsy report and our autopsy case, SMA-positive smooth muscle cells were ectopically scattered in the thickened intima of the MCAs [16]. VEGF acts on migration or proliferation of smooth muscle cells and promotes production of alcian blue-positive acid mucopolysaccharides [21, 22], which were observed in thickened intima of the previous and our autopsy cases. A previous case report describing pathology of a vulvar tumor has shown that some endothelial cells in the tumor were immunolabeled by anti-VEGF antibody along their luminal surface, and VEGF was thought to lead to myxomatous stroma of the tumor, which was stained by alcian blue [23]. Other cytokines that are upregulated in POEMS syndrome may be involved in cerebral vasculopathy. A report describing two patients with POEMS syndrome, Castleman disease, and recurrent ischemic strokes has speculated that interleukin-6 (IL-6) might play an important role in the occurrence of cerebral vasculopathy and ischemic stroke [10].

Large cerebral artery stenosis in POEMS syndrome occasionally shows mild improvement and might be at least partially reversible. In our study, improvement of stenotic lesions was observed in approximately one-fifth of patients who underwent follow-up MRA. Among the five patients with improved stenosis on follow-up MRA, improvement of all lesions was noted in two and of only one lesion in the remaining three. No patient had improved cerebral artery occlusion. In a case of POEMS syndrome with multiple cerebral artery stenoses/occlusions, mild improvement of stenotic lesions has been described [24]. These indicate a heterogeneous mechanism of cerebral vasculopathy in POEMS syndrome; irreversible structural changes of large cerebral arteries and reversible changes, such as cytokine-mediated vasoconstriction. Cases of POEMS syndrome with vasospastic angina have been reported [25, 26]. In one case, coronary vasospasm was induced by intracoronary injection of acetylcholine, and functional disturbance of the vascular endothelium associated with increased IL-6 levels is speculated as an underlying mechanism of coronary vasospasm [26]. Pulmonary hypertension has also been reported as a reversible vasculopathy in POEMS syndrome [27]. Factors that combine to produce increased vascular resistance (vasoconstriction, vascular wall remodeling, and thrombosis in situ), associated with overproduction of cytokines (IL-1β, IL-6, tumor necrosis factor-α, and VEGF), have been implicated in the pathogenesis of pulmonary hypertension in POEMS syndrome, as suggested for primary pulmonary hypertension [27, 28]. Pro-inflammatory cytokines, such as IL-1β, IL-6, and tumor necrosis factor-α, which have been shown to be activated in POEMS syndrome [29], are reportedly associated with cerebral vasospasm after subarachnoid hemorrhage [30, 31]. However, the temporal changes of cerebral artery stenosis/occlusion in POEMS syndrome have not been fully clarified. Further prospective longitudinal studies are needed to elucidate the background mechanism of the reversible nature of cerebral vasculopathy in POEMS syndrome.

There are several limitations in our study. First, this was a retrospective study and did not include consecutive patients during the study period; the cerebral vessels were not assessed in some patients. Second, some variables were reported to be associated with increased risk of cerebral infarction in POEMS syndrome, such as elevated blood platelet count and evidence of plasma cell proliferation on bone marrow biopsy [5]. Since elevated blood platelet count seems to be associated with increased risk of cerebral infarction by having an important role in thrombus formation [11], it is unlikely that elevated blood platelet count is involved in the structural change of blood vessels. Third, owing to the retrospective nature of this study, the imaging modalities or field strength of MRA were not consistent (including CTA, 1.5T MRA, and 3T MRA), and the time period from initial MRA to follow-up MRA varied among the subjects. Although differences in imaging modality and field strength of MRA can affect the visibility of the intracranial arteries [32], CTA was performed in only one patient; further, there was no significant difference in the type of MRI system (1.5T and 3T) between stenosis/occlusion and no stenosis/occlusion groups in this study. Finally, pro-inflammatory cytokines, such as tumor TNF-α, IL-6, and IL-12, which are upregulated in POEMS syndrome, were not evaluated, and this should be performed in future studies.

## Conclusions

In conclusion, stenosis or occlusion of the cerebral arteries is seen in approximately half of patients with POEMS, as vasculopathy is associated with the disease activity of POEMS syndrome. Cerebral vasculopathy in POEMS syndrome might be a heterogeneous condition with reversible nature and irreversible vascular structural changes presumably mediated by elevated inflammatory cytokines.

## Supplementary information

Additional file 1: **Table S1.** Clinical data and MRA findings of cases with improved stenosis/occlusion on follow-up MRA. (xls.)

## Data Availability

The complete data are available from the corresponding author on reasonable request.

## Abbreviations

POEMS: Polyneuropathy, organomegaly, endocrinopathy, M-protein, and skin changes
VEGF: Vascular endothelial growth factor
CTA: Computed tomography angiography
MRA: Magnetic resonance angiography
ACA: Anterior cerebral artery
MCA: Middle cerebral artery
VA: vertebral artery
PCA: Posterior cerebral artery
ICA: Internal carotid artery
BA: Basilar artery
SMA: Smooth muscle actin
IL-6: Interleukin-6
