# Supplementary material for: Cerebral large artery stenosis and occlusion in POEMS syndrome
Source: BMC Neurol. 2021 Jun 24;21:239. doi: 10.1186/s12883-021-02260-2 (PMC8223276; doi:10.1186/s12883-021-02260-2)
Supplement: Supplementary file 1 — Table S1. Clinical data and MRA findings of cases with improved stenosis/occlusion on follow-up MRA. (xls.) [file 12883_2021_2260_MOESM1_ESM.docx]

Research Article, *BMC Neurology*

**Cerebral large artery stenosis and occlusion in POEMS syndrome**

Atsuhiko Sugiyama, MD, PhD,^a^ Hajime Yokota, MD, PhD,^b^ Sonoko Misawa, MD, PhD,^a^ Hiroki Mukai, MD, PhD,^c^ Yukari Sekiguchi, MD, PhD,^d^ Kyosuke Koide, MD,^a^ Tomoki Suichi, MD, PhD,^a^ Jun Matsushima, MD, PhD,^e,f^ Takashi Kishimoto, MD, PhD,^g^ Zen-ichi Tanei, MD, PhD,^h^ Yuko Saito, MD, PhD,^h^ Shoichi Ito, MD, PhD,^a,i^ Satoshi Kuwabara, MD, PhD^a^

^a^ Department of Neurology, Graduate School of Medicine, Chiba University, Chiba, Japan

^b^ Department of Diagnostic Radiology and Radiation Oncology, Graduate School of Medicine, Chiba University, Chiba, Japan

^c^ Department of Radiology, Chiba University Hospital, Chiba, Japan

^d^ Department of Neurology, JR Tokyo General Hospital, Tokyo, Japan

^e^ Department of Pathology, Dokkyo Medical University, Saitama Medical Center, Saitama, Japan

^f^ Department of Diagnostic pathology, Graduate School of Medicine, Chiba University, Chiba, Japan

^g^ Department of Molecular Pathology, Graduate School of Medicine, Chiba University, Chiba, Japan

^h^ Department of Pathology and Laboratory Medicine, National Center of Neurology and Psychiatry, Tokyo, Japan

^i^ Department of Medical Education, Graduate School of Medicine, Chiba University, Chiba, Japan

| **Additional file 1: Table S1. Clinical data and MRA findings of cases with improved stenosis/occlusion on follow-up MRA** | | | | | | | | | | | |  |  |  |
| --- | --- | --- | --- | --- | --- | --- | --- | --- | --- | --- | --- | --- | --- | --- |
| Case | Sex | Initial MRA | | |  | Follow-up MRA | | | Follow-up period (years) | Treatment | |  | VEGF (pg/mL) | |
|  |  | Age | Field strength | Findings |  | Age | Field strength | Findings |  | Before initial MRA | Follow-up period |  | At initial MRA | At follow-up MRA |
| 1 | F | 35 | 1.5 | Grade 1: Bil. ICA, Bil. PCA |  | 41 | 1.5 | None | 6 | Thalidomide + Dex | Thalidomide + Dex ASCT |  | 3060 | 490 |
|  |  |  |  |  |  |  |  |  |  |  |  |  |  |  |
| 2 | M | 61 | 3 | Grade 1: Bil. ACA, Rt. MCA Grade 2: Rt. ICA |  | 61 | 3 | Grade 1: Rt. ACA, Rt. MCA Grade 2: Rt. ICA | 0.6 | None | Thalidomide + Dex |  | 16400 | 1410 |
|  |  |  |  |  |  |  |  |  |  |  |  |  |  |  |
| 3 | M | 58 | 1.5 | Grade 1: Rt. ACA, Lt. MCA |  | 60 | 1.5 | None | 1.9 | None | Thalidomide + Dex  Renalidomide + Dex |  | 6250 | 680 |
|  |  |  |  |  |  |  |  |  |  |  |  |  |  |  |
| 4 | F | 34 | 1.5 | Grade 1: Rt. ICA, Rt. ACA |  | 37 | 1.5 | Grade 1: Rt. ACA | 2.5 | None | Thalidomide + Dex Renalidomide + Dex |  | 11400 | 850 |
|  |  |  |  |  |  |  |  |  |  |  |  |  |  |  |
| 5 | M | 68 | 1.5 | Grade 1: Rt. MCA, Bil. PCA |  | 70 | 1.5 | Grade 1: Rt. MCA, Rt. PCA | 1.8 | None | Thalidomide + Dex  Renalidomide + Dex |  | 7170 | 736 |
|  |  |  |  |  |  |  |  |  |  |  |  |  |  |  |
| MRA, magnetic resonance angiography; Bil, bilateral; Rt, right; Lt, left; ICA, internal carotid artery; ACA, anterior cerebral artery; MCA, middle cerebral artery; PCA, posterior cerebral artery; Dex, dexamethasone; ASCT, autologous peripheral blood stem-cell transplantation | | | | | | | | | | | | | | |
